# Perceptions, facilitators, and barriers regarding use of the injury prevention exercise programme *Knee Control* among players and coaches in youth floorball: a cross-sectional survey study

**DOI:** 10.1186/s13102-023-00660-0

**Published:** 2023-04-13

**Authors:** Ida Åkerlund, Sofi Sonesson, Hanna Lindblom, Markus Waldén, Martin Hägglund

**Affiliations:** 1 Unit of Physiotherapy, Division of Prevention, Rehabilitation and Community Medicine, Department of Health, Medicine and Caring Sciences, Linköping University, s-581 83 Linköping, Sweden; 2 Sport Without Injury ProgrammE (SWIPE), Department of Health, Medicine and Caring Sciences, Linköping University, Linköping, Sweden; 3 GHP Ortho & Spine Center Skåne, Malmö, Sweden

**Keywords:** Athletic injuries, Implementation, Injury prevention, Neuromuscular training, Self-efficacy, Team sports

## Abstract

**Background:**

Youth participation in team ball sports is associated with a risk of both acute and gradual onset injuries but today there are several efficacious injury prevention exercise programmes (IPEPs). However, there is limited research about how to implement those programmes and the perceived barriers and facilitators among end-users.

**Objective:**

To investigate perceptions of the IPEP *Knee Control* and facilitators and barriers to programme use among coaches and youth floorball players, and explore factors associated with planned maintenance of *Knee Control*.

**Methods:**

This cross-sectional study is a sub-analysis of data from the intervention group of a cluster randomised controlled trial. Perceptions about *Knee Control* and facilitators and barriers to programme use were evaluated with surveys pre-intervention and post-season. 246 youth floorball players aged 12–17 years, and 35 coaches that reported no use of IPEPs during the preceding year were included. Descriptive statistics and univariate and multivariate ordinal logistic regression models were undertaken with the dependent variables: coaches’ planned maintenance and players’ opinions of maintenance of *Knee Control.* Independent variables were perceptions, facilitators and barriers regarding use of *Knee Control* and other potential influencing factors.

**Results:**

88% of the players believed that *Knee Control* can reduce injury risk. Common facilitators to *Knee Control* use among coaches were support, education and high player motivation, and common barriers were that injury prevention training was time-consuming, lack of space to execute the exercises and lack of player motivation. Players who planned to maintain use of *Knee Control* had higher outcome expectancies and belief in one’s ability to use *Knee Control* (action self-efficacy). Coaches who planned to maintain *Knee Control* had higher action self-efficacy and to a lesser extent considered that *Knee Control* takes too much time.

**Conclusions:**

Support, education, and high player motivation are key facilitators, while lack of time and space for injury prevention training and boring exercises are key barriers for coaches and players to use *Knee Control*. High action self-efficacy among coaches and players seems to be a prerequisite for maintained use of IPEPs.

**Supplementary Information:**

The online version contains supplementary material available at 10.1186/s13102-023-00660-0.

## Introduction

Floorball is played indoors using a plastic stick and light plastic ball on a 40 × 20 m pitch with five field players and one goalkeeper in each team. The sport involves fast running with sudden stops, accelerations/decelerations and sharp changes of direction [1]. Team ball sports participation is associated with a risk of both acute and gradual onset (overuse) injuries, not least in adolescent players [2, 3]. Injury prevention exercise programmes (IPEPs) have been successful in preventing both acute and gradual onset injuries in different team ball sports [4–6], especially among teams/players exhibiting high compliance with the intervention [7, 8]. The Swedish IPEP *Knee Control* has been shown to reduce the risk of anterior cruciate ligament (ACL) injuries by 64% in female youth football players [9] and the risk of overall acute injuries by 45% in male and female youth floorball players [10]. A dose–response relationship, with a greater reduction in injury risk with higher IPEP dose, was seen in both football and floorball [11, 12].

Even though IPEPs have been efficacious in preventing injuries in structured randomised controlled trials (RCTs), it is still challenging to achieve widespread adoption and maintenance in a real-world context [13]. Implementation is defined as the extent to which an IPEP is delivered as intended [14]. A better understanding of the context [14], how the intervention is perceived by its users and how it influences end-user behaviour is important to succeed with implementation [15, 16]. Maintenance of an IPEP can be defined as “the extent to which the intervention is sustained over time” [14], which is central to achieve long-term IPEP effectiveness [16]. To maximise uptake and maintenance of the IPEP, the implementation strategy should focus on behaviour change in coaches [17]. The Health Action Process Approach (HAPA) model, a theory of health behaviour change, comprises two phases. A motivation phase, where risk perceptions, outcome expectancies and action self-efficacy are important constructs; and a volitional phase, where intention must be translated into action [18].

Various facilitators and barriers can affect use of an IPEP [19]. Implementation barriers to programme use among coaches include low player motivation, lack of time and lack of knowledge among coaches about how to implement the programme, and IPEP-related barriers, such as being time-consuming and lacking sport-specificity [17, 20–24]. Implementation facilitators among coaches include observational learning (i.e., support from others), adequate resources (enough space, videos, apps), belief that the programme enhances performance, and IPEP-related factors, such as using a ball or sport-specific skills training, and appropriate progression and variation of exercises [17, 18, 20–22, 24, 25]. Player-perceived barriers to implementation are boring exercises, lack of ball work and reduced sport-specific training time, and examples of player-perceived facilitators are acceptance of the IPEP, motivation and a belief that the IPEP can prevent injuries [17, 22–24]. Previous studies have mainly been carried out in a youth football context but some also in youth rugby and elite handball.

There is limited research exploring the sport-specific contextual factors that influence programme implementation in floorball [13, 26]. This study aimed to investigate perceptions of *Knee Control* and facilitators and barriers to programme use among coaches and youth floorball players, and to explore factors associated with planned maintenance of *Knee Control*.

## Methods

### Study design and participants

This cross-sectional study is a sub-analysis of data from the intervention group in a two-armed cluster RCT that evaluated the preventive effect of the Swedish IPEP *Knee Control* (Knäkontroll, SISU Idrottsböcker, Sweden, 2005) on injuries among male and female floorball players at youth community level in two districts of Sweden during 2017–2018 [10]. The overall study design, the main results of the RCT and the association between intervention compliance and injury rates have been reported previously [10, 12]. To be included in the RCT, the players had to be 12–17 years old, not used any IPEP regularly in the last year, and have had ≥ 2 scheduled team training sessions per week over the season. The RCT was prospectively registered with Clinical Trials (NCT03309904) (submitted 10 October, posted 16 October 2017).

### Intervention

The intervention consisted of a standardised running warm-up (including change of direction and acceleration/deceleration drills), followed by *Knee Control* exercises with three sets of 8–15 repetitions for each exercise (10–15 min). *Knee Control* consists of six main exercises (one-legged knee squat, pelvic lift, two-legged knee squat, the bench, the lunge and jump/landing technique) (Additional file 1). The coaches were told to use the running warm-up and *Knee Control* before every single training session throughout the 26-week season. The exercises were often performed in a corridor or in a locker room before the team had access to the floorball court, and the stick and ball were used in some of the exercises.

The intervention group coaches plus 1–2 players per team were invited to a three-hour implementation workshop at the beginning of the floorball season (September 2017). Information regarding injury risk in floorball and the injury preventive effects of *Knee Control* in football were presented. During a practical session, the coaches and player representatives were instructed about the correct execution of the running warm-up and *Knee Control* exercises. The programme was made available to coaches through written instructions with explanatory text, pictures and videos. The workshop structure, consisting of feedback and practical advice, aimed to increase coaches’ self-efficacy (belief in one’s ability to use *Knee Control*) and exercise fidelity (performing exercises with correct technique) with the programme.

### Player and coach pre-intervention and post-season surveys

The majority of survey questions were based on the HAPA model [18] Players’ and coaches’ perceptions, in terms of risk perceptions, outcome expectancies (about injury risk and player performance in floorball), action self-efficacy, behavioural intention (e.g. planned maintenance of the programme) (Table 1), appraisals of *Knee Control* and perceived facilitators and barriers to *Knee Control* use (Table 2) were studied by means of pre-intervention (Additional files 2 and 3) and/or post-season surveys (Additional files 4 and 5). The surveys were designed based on previously used questionnaires that have been face validated [22, 24], and subsequently adapted by two of the authors to a Swedish floorball context.

**Table 1:** Pre-intervention and post-season player and coach survey questions aligned with relevant HAPA constructs

HAPA construct and question	Pre-intervention coach survey	Post-season coach survey	Pre-intervention player survey	Post-season player survey
*Risk perceptions (motivational phase)*				
What is your opinion about the overall risk of injury in floorball? low–7 high) [24]	X			
I expect I will sustain an injury sometime during this season… (1 likely–7 unlikely) [24]			X	
*Outcome expectancies (motivational phase)*				
In general, how preventable do you think floorball injuries are? (1 not preventable–7 preventable) [24]	X	X		
In your opinion, what would happen to a floorball player’s overall risk of injury if he/she participated in injury prevention training? (1 increase–7 decrease) [24]	X	X		
What do you think would happen to a floorball player’s performance if he/she did injury prevention training regularly? (1 decrease–7 increase)	X	X		
Many sports injuries are preventable with the help of training or protective equipment… (1 false–7 true) [24]			X	
After training *Knee Control* this season, I think my risk of injury has… (1 increased–7 decreased) [24]				X
After training *Knee Control* this season, I have become faster, stronger and developed better balance…(1 false–7 true)				X
*Action self-efficacy (motivational phase)*				
My knowledge about preventing injuries in floorball is… (1 poor–7 good) [24]	X	X		
My practical ability to use *Knee Control* with my team is… (1 poor–7 good) [24]		X		
I have listened to my coach’s instructions on how to do the *Knee Control* exercises…(1 little–7 much) [24]				X
I have been able to do all the exercises in the *Knee Control* programme correctly… (1 unsure–7 sure) [24]				X
I have made 100% effort when we practised the *Knee Control* exercises… (1 false–7 true) [24]				X
*Behavioural intention*				
Are you planning to prioritise injury prevention training in the form of *Knee Control* next season? (1 uncertain–7 certain) [24]		X		
If my team uses *Knee Control* next season, I think it is… (1 bad–7 good) [24]				X

“Extremely” to be added to all anchors in the Likert scale, e.g., 1 extremely low–7 extremely high

Abbreviations: HAPA, the Health Action Process Approach model

**Table 2:** Post-season player and coach questions about appraisal of *Knee Control* and perceived facilitators and barriers to *Knee Control* use

Questions
Coach
Appraisal of *Knee Control*
*Knee Control* is floorball-specific… (1 false–7 true) [20]
*Knee Control* takes too much time… (1 false–7 true) [20]
*Knee Control* contains appropriate variation and progression for our team… (1 false–7 true) [20]
*Knee Control* can be used over several seasons in our team… (1 false–7 true) [20]
Facilitators and barriers
The following facilitators can help me perform *Knee Control* with my team at every training session next season (open answer)
The following barriers can make it difficult for me to perform *Knee Control* with my team at every training session next season (open answer)
Player
Appraisal of *Knee Control*
What did you like about *Knee Control* (9 closed answer options, several choices were possible)
What did you not like about *Knee Control* (9 closed answer options, several choices were possible)

“Extremely” to be added to all anchors in the Likert scale, 1 extremely false–7 extremely true

### Data collection

The pre-intervention surveys were sent out to all players and coaches prior to the implementation workshop. Coaches who had not answered the survey before the workshop got a reminder and did so immediately after, prior to intervention start. All players responded before the intervention period started. The post-season surveys were sent out exclusively to the players and coaches in the intervention group after the competitive season. Reminders were sent out if the player or coach did not respond. An online secure software (esMakerNX3 V3) was used to manage the web-based surveys.

Players also answered a weekly web-based survey that asked questions including the occurrence of any injury during the past week using the Oslo Sports Trauma Research Centre’s Overuse Injury Questionnaire [27]. Coaches used a standard individual player attendance form and also documented whether the team had completed *Knee Control* (yes/no) which enabled the calculation of player compliance with *Knee Control*. Players were allocated to three expedient compliance groups based on their average weekly use of *Knee Control*: high dose (≥ 2 *Knee Control* sessions per week), intermediate dose (≥ 1 to < 2 *Knee Control* sessions per week), and low dose (< 1 *Knee Control* session per week) [12].

### Statistics

Most questions and statements in the pre-intervention and post-season surveys were answered using a 7-point Likert scale. Descriptive statistics were used for demographic data, perceptions of *Knee Control* and facilitators and barriers for *Knee Control* use. The Wilcoxon signed rank sum test was used for comparisons of coaches’ injury prevention expectancies between pre-intervention and post-season responses.

Coaches’ planned maintenance and players’ opinions of maintenance of *Knee Control* were chosen as the dependent variable in an ordinal logistic regression analysis because maintained IPEP use is central to achieving long-term IPEP effectiveness [16]. The independent variables were chosen to include perceptions about and facilitators and barriers for *Knee Control* use and other potential influencing factors, such as if the player had sustained an injury during the season, and *Knee Control* dose during the season.

For players, a generalised ordinal logistic regression with simple and multiple models was used to study the association between their opinion about the maintenance of *Knee Control* – “If my team uses *Knee Control* next season, I think it is…” (1 extremely bad–7 extremely good) – and potential influencing factors. All variables with a p-value < 0.10 were entered into a multiple ordinal regression model and then a stepwise backward elimination procedure was applied with a p-value > 0.10 being the criterion for variable elimination. The multiple analyses were adjusted for sex.

For coaches, simple and multiple binary logistic regression models were used. The dependent variable: “Are you planning to prioritise injury prevention training in the form of *Knee Control* next season?” (1 extremely uncertain–7 extremely certain) was divided into negative (1–2), neutral (3–5) and positive (6–7) due to the small sample size and the small distribution of answers in the variable (range of answer options from 4 to 7 on the Likert scale). A binary model was used because only the neutral and positive categories were represented in the dependent variable. The same procedure as for players regarding the selection and elimination of independent variables in the multiple models was used, except that only post-season variables were selected when the same questions were included in both pre-intervention and post-season surveys. The multiple analyses were adjusted for being a coach for a male/female team.

A value of 7 on the Likert scale was set as the reference in all calculations. No imputation was made for missing data. All analyses were performed using SPSS statistical software for Windows (v27; IBM, New York), and a p-value < 0.05 was considered to be significant. No a priori sample size calculation was made for these post-hoc exploratory sub-analyses.

## Results

### Participants

In total, 31 teams (8 female) with 301 players (107 female) in 17 clubs, and 48 coaches (12 for female teams) were included in the intervention arm of the cluster RCT. Of these, 246 players (82%, 93 female) and 35 coaches (73%, 12 for female teams) answered both pre-intervention and post-season surveys and are included in this sub-analysis. Mean player age was 13.7 ± 1.1 years (male 13.5 ± 0.9, female 13.9 ± 1.4).

### Risk perceptions, outcome expectancies and action self-efficacy regarding *Knee Control* use

Players’ risk perceptions and outcome expectancies are presented in Table 3. Players had strong belief in their ability to use *Knee Control* (action self-efficacy) (Table 3), and male and female players reported similar views (Table 1 and Additional file 6). Coaches’ risk perceptions and outcome expectancies pre-intervention are presented in Table 4. Action self-efficacy regarding knowledge about how to prevent injuries increased during the season (Table 4). Coaches for female teams reported increased knowledge from pre- to post-intervention (median 3.5 to 5 on a 1–7 Likert scale, respectively). Coaches for male teams reported increased knowledge from pre- to post-intervention (median 4 to 5 on a 1–7 Likert scale, respectively). Data separated for coaches for male and female teams are presented in Table 2 in Additional file 6.

**Table 3:** Players' *(n* = *246)* risk perceptions, outcome expectancies and action self-efficacy regarding *Knee Control* use

**Questions**	Total
Risk perceptions	
*Pre-intervention*	
I expect I will sustain an injury sometime during this season… (1 likely–7 unlikely)	4 (2)^a^
Outcome expectancies	
*Pre-intervention*	
Many sports injuries are preventable with the help of training or protective equipment… (1 false–7 true)	6 (1)^b^
*Post-season*	
After training *Knee Control* this season, I think my risk of injury has… (1 increased–7 decreased)	5 (2)^c^
After training *Knee Control* this season, I have become faster, stronger and developed better balance… (1 false–7 true)	5 (1)^c^
Action self-efficacy	
*Post-season*	
I have listened to my coach’s instructions on how to do the *Knee Control* exercises… (1 little–7 much)	6 (1)^c^
I have been able to do all the exercises in the *Knee Control* programme correctly… (1 unsure–7 sure)	6 (2)^c^
I have made 100% effort when we practised the *Knee Control* exercises… (1 false–7 true)	6 (1)^c^

All results are presented as median with interquartile range in brackets. “Extremely” to be added to all anchors in the Likert scale, e.g., 1 extremely likely–7 extremely unlikely

^a^Missing for 10 players

^b^Missing for 11 players

^c^Missing for 1 player

**Table 4:** Coaches’ *(n* = *35)* risk perceptions, outcome expectancies, action self-efficacy and appraisal of *Knee Control*

**Questions**	Pre-intervention	Post-season	P-value
*Risk perceptions*			
What is your opinion about the overall risk of injury in floorball? (1 low–7 high)	4 (2)		
*Outcome expectancies*			
In general, how preventable do you think floorball injuries are? (1 not preventable–7 preventable)	6 (1)	6 (1)	0.472
In your opinion, what would/has happen/ed to a floorball player’s overall risk of injury if he/she participated in injury prevention training? (1 increase–7 decrease)	5 (4)	5 (3)	0.796
What do you think would/has happen/ed to a floorball player's performance if he/she did injury prevention training regularly? (1 decrease–7 increase)	5 (1)	5 (2)	0.226
*Action self-efficacy*			
My knowledge about preventing injuries in floorball is…(1 poor–7 good)	4 (2)	5 (1)	**0.008**
My practical ability to use *Knee Control* with my team is…(1 poor–7 good)		5 (1)	
*Appraisal of Knee Control*			
*Knee Control* is floorball specific…(1 false–7 true)		5 (2)^a^	
*Knee Control* takes too much time…(1 false–7 true)		4 (3)	
*Knee Control* contains appropriate variation and progression for our team…(1 false–7 true)		5 (1)	
*Knee Control* can be used over several seasons in our team…(1 false–7 true)		6 (1)	

Bold values indicate statistically significant results

All results are presented as median with interquartile range in brackets. “Extremely” to be added to all anchors in the Likert scale, e.g., 1 extremely not preventable–7 extremely preventable. P-values refer to comparison between coaches’ pre-intervention and post-season survey responses

^a^Missing for 1 coach

### Appraisals of *Knee Control* and facilitators and barriers to programme use

Players stated as positive aspects of *Knee Control* that the programme can reduce injury risk (88%), that they became better at performing *Knee Control* during the season (72%), and that the exercises can be performed together in pairs or with the whole team (48%). Negative aspects were that they had less time for floorball training (43%), the exercises were boring (40%) or that they experienced pain during the exercises (24%). A complete list of the players’ appraisals of *Knee Control* is shown in Table 3 and Additional file 6. Coaches appraised that *Knee Control* could be used over several seasons (Table 4).

The most commonly reported facilitators among coaches were support and education, high player motivation, further development of *Knee Control*, more training time and better resources (documentation of *Knee Control* and material). The most common barriers were that injury prevention training was time-consuming, a lack of space to do the exercises and lack of player motivation.

### Players’ opinion of maintenance and coaches’ planned maintenance of *Knee Control* use and associations with potential influencing factors

Overall, among players a significant positive association was found between views of *Knee Control* maintenance and post-season outcome expectancies (about injury risk and player performance) and belief in one’s ability to use *Knee Control* (action self-efficacy). Table 5 summarises the results of the ordinal logistic regression analysis of independent variables associated with players’ opinions of the maintenance of *Knee Control*. For coaches, a positive association was seen between the planned maintenance of *Knee Control* and action self-efficacy post-season, and there was a negative association with the appraisal that *Knee Control* takes too much time (Table 6).

**Table 5:** Ordinal logistic regression models exploring the associations between players’ *(n* = *246)* opinion of maintenance of *Knee Control* and potential influencing factors

Variable	Simple ordinal logistic regression			Multiple ordinal logistic regression		
	Regression coefficient (95% CI)	P-value	Odds ratio (95% CI)	Regression coefficient (95% CI)	P-value	Odds ratio (95% CI)
*Demographics*						
Age	− 0.02 ( − 0.21 to 0.18)	0.877	0.99 (0.81–1.20)			
Sex (male 0/female 1, reference)	− 0.53 ( − 0.99 to − 0.07)	**0.025**	0.59 (0.37–0.94)	− 0.49 ( − 0.96 to − 0.02)	**0.042**	0.61 (0.38–0.98)
Earlier experience of *Knee Control*		0.472				
Don’t know	− 0.54 ( − 1.38 to 0.30)	0.204	0.58 (0.25–1.34)			
No	− 0.01 ( − 0.65 to 0.63)	0.981	0.99 (0.52–1.88)			
Yes, sometimes during the last year	− 0.23 ( − 0.92 to 0.47)	0.521	0.80 (0.40–1.59)			
Yes, regularly during the last year	Reference					
*Risk perceptions pre-intervention*						
I expect I will sustain an injury sometime during this season…(1 likely–7 unlikely)	0.06 ( − 0.11 to 0.22)	0.507	1.06 (0.90–1.25)			
*Outcome expectancies*						
Pre-intervention: Many sports injuries are preventable with the help of training or protective equipment… (1 false–7 true)	0.12 ( − 0.12 to 0.36)	0.348	1.12 (0.88–1.44)			
Post-season: After training *Knee Control* this season, I think my risk of injury has… (1 increased–7 decreased)	0.62 (0.40–0.83)	** < 0.001**	1.90 (1.50–2.29)	0.56 (0.35–0.78)	** < 0.001**	1.75 (1.41–2.18)
Post-season: After training *Knee Control* this season, I have become faster, stronger and developed better balance… (1 false–7 true)	0.72 (0.51–0.94)	** < 0.001**	2.06 (1.66–2.56)	0.52 (0.30–0.74)	** < 0.001**	1.68 (1.34–2.10)
*Action self-efficacy*						
I have listened to my coach’s instructions on how to do the *Knee Control* exercises… (1 little–7 much)	0.53 (0.35–0.72)	** < 0.001**	1.71 (1.42–2.05)	0.24 (0.02–0.47)	**0.032**	1.28 (1.02–1.60)
I have been able to do all the exercises in the *Knee Control* programme correctly… (1 unsure–7 sure)	0.40 (0.22–0.58)	** < 0.001**	1.49 (1.24–1.78)			
I have made 100% effort when we practised the *Knee Control* exercises… (1 false–7 true)	0.62 (0.42–0.83)	** < 0.001**	1.87 (1.52–2.29)	0.29 (0.03–0.54)	**0.027**	1.33 (1.03–1.72)
*Other potential influencing factors*						
Injury during season (no 0/yes 1)	− 0.14 ( − 0.58 to 0.31)	0.550	0.87 (0.56–1.36)			
*Knee Control* dose		**0.025**				
Low dose < 1 session per week	− 0.92 ( − 1.67 to − 0.18)	**0.015**	0.40 (0.19–0.84)			
Intermediate dose ≤ 1 to < 2 sessions per week	− 0.66 ( − 1.24 to − 0.08)	**0.027**	0.52 (0.29–0.93)			
High dose ≥ 2 sessions per week	Reference					

“Extremely” to be added to all anchors in the Likert scale, e.g., 1 extremely likely–7 extremely unlikely. A value of 7 is the reference for all variables with a Likert scale. Dependent variable: If my team uses *Knee Control* next season, I think it is… (1 bad–7 good). The multiple analyses are adjusted for sex. Variables with p < 0.10 in the simple analyses were included in the multiple analyses. Only variables with p < 0.10 in the multiple analyses are reported in the table. Bold values indicate statistically significant results

CI, confidence interval

**Table 6:** Binary logistic regression models exploring the associations between coaches’ *(n* = *35)* planned maintenance of *Knee Control* and potential influencing factors

Variable	Simple binary logistic regression			Multiple binary logistic regression		
	Regression coefficient (S.E.)	P-value	Odds ratio (95% CI)	Regression coefficient (S.E.)	P-value	Odds ratio (95% CI)
Coach for male/female team (male 0/female 1, reference)	0.29 (0.73)	0.689	1.34 (0.32–5.61)	− 0.01 (0.99)	0.921	0.91 (0.13–6.36)
*Risk perceptions*						
What is your opinion about the overall injury risk in floorball? (1 low–7 high)	0.49 (0.35)	0.164	1.63 (0.82–3.26)			
*Outcome expectancies*						
Pre-intervention, in general, how preventable do you think floorball injuries are? (1 not preventable–7 preventable)	1.79 (0.71)	0.012	5.99 (1.48–24.20)			
Post-season, in general, how preventable do you think floorball injuries are? (1 not preventable–7 preventable)	0.45 (0.40)	0.261	1.57 (0.72–3.46)			
Pre-intervention, in your opinion, what would happen to a floorball player’s overall risk of injury if he/she participated in injury prevention training? (1 increase–7 decrease)	0.23 (0.19)	0.239	1.26 (0.86–1.83)			
Post-season, in your opinion, what would happen to a floorball player’s overall risk of injury if he/she participated in injury prevention training? (1 increase–7 decrease)	0.27 (0.23)	0.249	1.31 (0.83–2.07)			
Pre-intervention, what do you think would happen to a floorball player’s performance if he/she did injury prevention training regularly? (1 decrease–7 increase)	0.66 (0.37)	0.073	1.94 (0.94–4.00)			
Post-season, what do you think would happen to a floorball player’s performance if he/she did injury prevention training regularly? (1 decrease–7 increase)	0.55 (0.39)	0.159	1.73 (0.81–3.72)			
*Action self-efficacy*						
Pre-intervention, my knowledge about preventing injuries in floorball is… (1 poor–7 good)	0.81 (0.40)	**0.041**	2.25 (1.03–4.90)			
Post-season, my knowledge about preventing injuries in floorball is… (1 poor–7 good)	1.18 (0.50)	**0.018**	3.26 (1.22–8.69)			
Post-season, my practical ability to use *Knee Control* with my team is… (1 poor–7 good)	1.73 (0.75)	**0.021**	5.66 (1.31–24.58)	1.81 (0.88)	**0.040**	6.01 (1.08–34.14)
*Appraisal of Knee Control*						
*Knee Control* is floorball specific… (1 false–7 true)	− 0.01 (0.26)	0.958	0.99 (0.60–1.63)			
*Knee Control* takes too much time… (1 false–7 true)	− 0.80 (0.29)	**0.006**	0.45 (0.25–0.79)	− 0.81 (0.33)	**0.014**	0.45 (0.23–0.85)
*Knee Control* contains appropriate variation and progression for our team… (1 false–7 true)	1.25 (0.70)	0.075	3.49 (0.88–13.79)			
*Knee Control* can be used over several seasons in our team… (1 false–7 true)	0.08 (0.37)	0.837	1.08 (0.52–2.24)			

“Extremely” to be added to all anchors in the Likert scale, e.g., 1 extremely low–7 extremely high. A value of 7 is the reference for all variables with a Likert scale. Dependent variable: are you planning to prioritise injury prevention training in the form of *Knee Control* next season? (1 uncertain–7 certain), divided into negative (1–2), neutral (3–5), positive (6–7). The multiple analyses are adjusted for coach for male/female team. Variables with p < 0.10 in the simple analyses were included in the multiple analyses. Only variables with p < 0.10 in the multiple analyses are reported in the table. Bold values indicate statistically significant results

S.E., standard error; CI, confidence interval

## Discussion

Our main findings were that players showed high action self-efficacy i.e., they believed in their ability to use *Knee Control*. Factors that were positively associated with players’ views about maintained *Knee Control* use were outcome expectancies and action self-efficacy post-season. The coaches reported facilitators for the implementation of *Knee Control* related to the IPEP, and to external factors such as high motivation among players and support and education regarding *Knee Control*. The perceived barriers among coaches were mainly of a practical nature, e.g., that injury prevention training was too time-consuming and a lack of space to do the exercises. For coaches, a positive association was seen between the planned maintenance of *Knee Control* and action self-efficacy post-season, and a negative association with the appraisal that *Knee Control* takes too much time.

Players and coaches were slightly positive, but not strongly convinced, that *Knee Control* would reduce the players’ risk of injury or increase player performance (median 5 on a 1–7 Likert scale), which seem to be relevant key factors to succeed with IPEP implementation [21].

Coaches perceived that their knowledge about preventing injuries and practical ability to use *Knee Control* were fairly good (median 5 on a 1–7 Likert scale). Even if the change pre- to post-intervention was statistically significant (P = 0.008), it was only a one step improvement from median 4 (neither good nor bad) to 5 (fairly good) which might be of questionable relevance in practical terms. This suggests that our efforts to increase knowledge about injury prevention and boost the coaches’ belief in their ability to use *Knee Control* in the pre-season implementation workshop was insufficient. A single workshop at the beginning of the season may not be enough to support coaches and this emphasises the importance of further coach support from peers, their club and sport associations [25]. To succeed with the uptake and long-term maintenance of an IPEP in community-level sports, it is crucial that sports associations take responsibility for supporting coaches and players during the entire implementation process.

Players’ appraisals of *Knee Control* can also be interpreted within the framework of facilitators and barriers to programme use, e.g., the appraisal that *Knee Control* can reduce injury risk is referred to as a facilitator in previous studies [22, 23]. The appraisals that they had less time for floorball training, that exercises were boring and that some players experienced pain during exercises can be seen as barriers to programme use and have previously been reported as such in a similar study in football [22]. These barriers will probably affect a player’s motivation to use IPEP and need to be considered in future studies and when implementing IPEPs. However, coaches also play an essential role in motivating their players to engage in injury prevention training. Player-perceived facilitators can also be seen as motivators for IPEP use, e.g., having role models in the form of famous coaches and players who advocate their use, use of sport-related exercises with variations (partner exercises) and progressions, and to inform about the purpose of the exercises and their injury preventive effect [28]. Information about player-perceived facilitators and how coaches can use them to motivate their players could be added to the pre-season implementation workshop.

Adequate resources (manpower, enough space, videos, apps), belief that the programme enhances performance, and IPEP-related facilitators such as using a ball or sport-specific skills training, along with appropriate progression and variation of exercises, have been reported as facilitators to programme implementation in team ball sports [17, 21, 22, 24, 25]. Coaches in this study identified many of the same facilitators but, in the open-ended questions, they also stated that support and education, high player motivation and further development of *Knee Control* were important factors. Developing the programme to include more exercise options, progressions and sport-specific exercises may both facilitate coach implementation of the IPEP and address the player barrier of perceiving the exercises as boring. The most common barriers reported by coaches were similar to the players’ perceived barriers, including that injury prevention training was too time-consuming and lack of player motivation. These barriers, along with lack of space to do the exercises, have been reported in several studies [17, 20–24] and indicate that the implementation of IPEPs faces the same challenges in many different contexts. A crucial next step is to find sport-specific solutions for how to use the information about facilitators and barriers to improve implementation in the context of this specific sport. It is also of interest to learn more about players’ perceptions and experiences of *Knee Control* to gather suggestions on sport-specific solutions from the end user’s point of view. A better understanding of how coaches’ perceptions match up with the perceptions of their players may help to inform *Knee Control* delivery strategies and maximise implementation.

Factors that are important for players’ planned maintained use of *Knee Control* included high outcome expectancies, such as a decreased injury risk and better performance after practising *Knee Control*. In addition, a stronger belief in their ability to use *Knee Control* was associated with positive views about maintaining the programme. Coaches who planned to continue using *Knee Control* during the next season perceived a greater ability to use the programme and that it was not too time-consuming. High action self-efficacy, i.e., a strong belief in one’s ability to use *Knee Control*, seems to be a key factor for both players’ and coaches’ maintenance of the programme. This is also described in HAPA as a major predictor of intention to instigate health behaviour change during the motivation phase [29]. Even where players and coaches have good intentions to maintain *Knee Control*, this does not necessarily lead to action. Therefore, a long-term follow-up on the maintenance of *Knee Control* among youth floorball teams would be valuable.

This study has some limitations. Firstly, the surveys have not undergone a formal validation process, beyond face validity. Our experience is that they worked well for exploratory purposes; the questions are quite straightforward and should not leave much room for interpretation. However, under- or over-estimated assessments cannot be ruled out due to imprecision in the measurements. Three questions in the player pre- or post-season survey had the anchors extremely true/false in the 7-point Likert scale. Even though, the same descriptive information was used for the scale as in McKay et al. [24], the questions may have been difficult to understand and hence interpreted differently. Coaches who had not answered the pre-intervention survey before the implementation workshop may have been influenced by the contents of the workshop. Another limitation was that the coaches were part of an RCT which may have influenced their responses, for instance adoption of the programme was encouraged and supported as part of the study protocol. We studied coaches’ intention to maintain programme use, in future research actual maintenance of *Knee Control* use would be interesting to study. At last, the small number of coaches makes the data less robust, particularly in the regression analyses, and this must be taken into account when interpreting the results.

## Conclusions

Support, education, and high player motivation are key facilitators, while lack of time and space for injury prevention training and boring exercises are key barriers for coaches and players to use *Knee Control*. High action self-efficacy among coaches and players seems to be a prerequisite for maintained use of IPEPs. Study findings suggest that facilitators and barriers are important to address in order to achieve large scale implementation.

## Supplementary Information

**Additional file 1**. Details of the running warm-up programme and *Knee Control* injury prevention exercise programme (IPEP) used in the intervention group.

**Additional file 2**. Pre-intervention player survey. The survey in its entirety, not all questions are relevant in this paper

**Additional file 3**. Pre-intervention coach survey. The survey in its entirety, not all questions are relevant in this paper

**Additional file 4**. Post-season player survey. The survey in its entirety, not all questions are relevant in this paper

**Additional file 5**. Post-season coach survey. The survey in its entirety, not all questions are relevant in this paper

**Additional file 6**. **Tables S1-S3**. Sex-separated results for both players and coaches.

## Data Availability

The data that support the findings of this study are available from the corresponding author upon reasonable request.

## Abbreviations

IPEP: Injury prevention exercise programme
ACL: Anterior cruciate ligament
RCT: Randomised controlled trail
HAPA: The health action process approach
S.E.: Standard error
CI: Confidence interval
