# Supplementary material for: Perceptions, facilitators, and barriers regarding use of the injury prevention exercise programme Knee Control among players and coaches in youth floorball: a cross-sectional survey study
Source: BMC Sports Sci Med Rehabil. 2023 Apr 13;15:56. doi: 10.1186/s13102-023-00660-0 (PMC10103405; doi:10.1186/s13102-023-00660-0)
Supplement: Supplementary file 1 — Additional file 1. Details of the running warm-up programme and Knee Control injury prevention exercise programme (IPEP) used in the intervention group. [file 13102_2023_660_MOESM1_ESM.docx]

| Additional file 1. Details of the running warm-up programme and *Knee Control* injury prevention exercise programme (IPEP) used in the intervention group | | |
| --- | --- | --- |
| Exercise | **Instructions** | **Sets repetitions/duration** |
| Running exercises | Course consists of 6-10 pairs of parallel cones, approximately 10 m wide and 20 m long. To be performed before each training session and match. Ensure good hip-knee-foot alignment during the running exercises | 5 min |
| Running | Straight ahead |  |
| Sideways jumps | With bent knees and low center of gravity |  |
| Running | Slalom, with change of direction |  |
| Sideways jumps | Jump with body contact; shoulder to shoulder |  |
| Running | Alternate forwards and backwards running, with decelerations and accelerations |  |

| One legged knee squat | Slow movement with smooth turn, stable hip with horizontal pelvic position and non-supporting foot in front of the body with slightly flexed hip and knee. Knee flexion in alignment with the foot. | |
| --- | --- | --- |
| Level A | Hands on hips  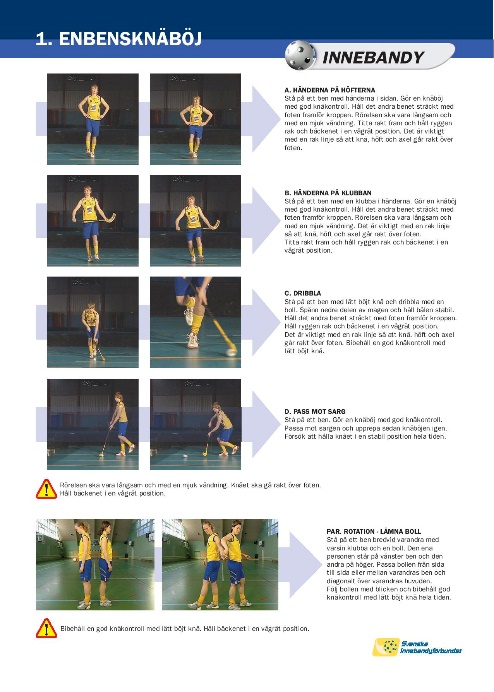 | 3×8-15 reps |
| Level B | Hold a stick  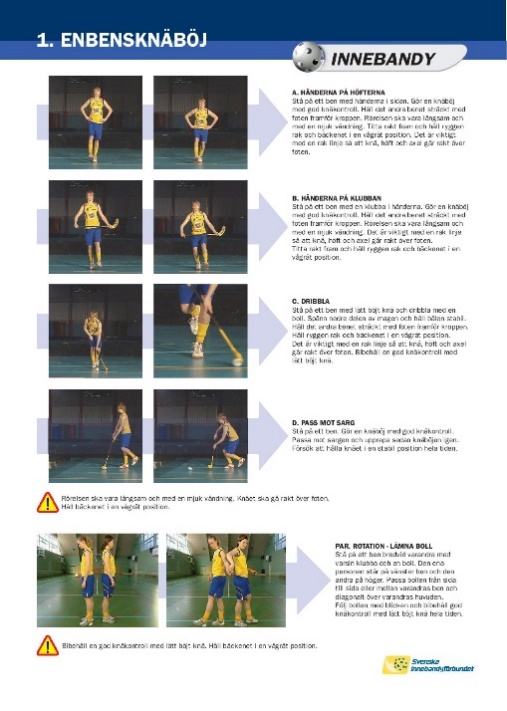 | 3×8-15 reps |
| Level C | Dribble in front of you  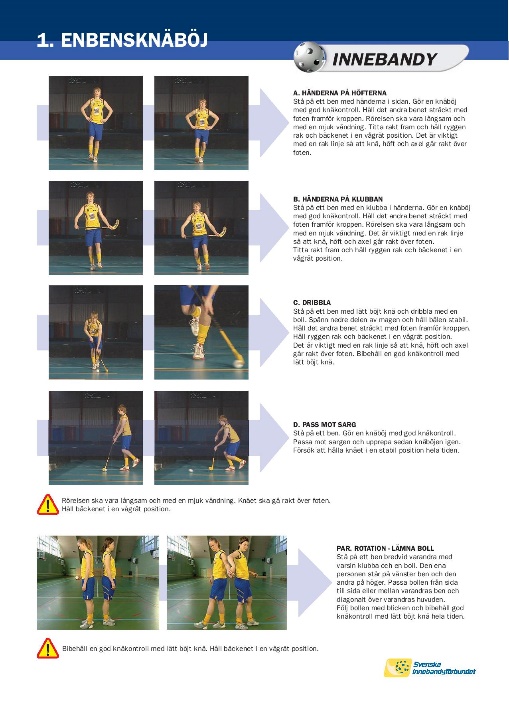 | 3×8-15 reps |
| Level D | Pass the ball against the board or wall  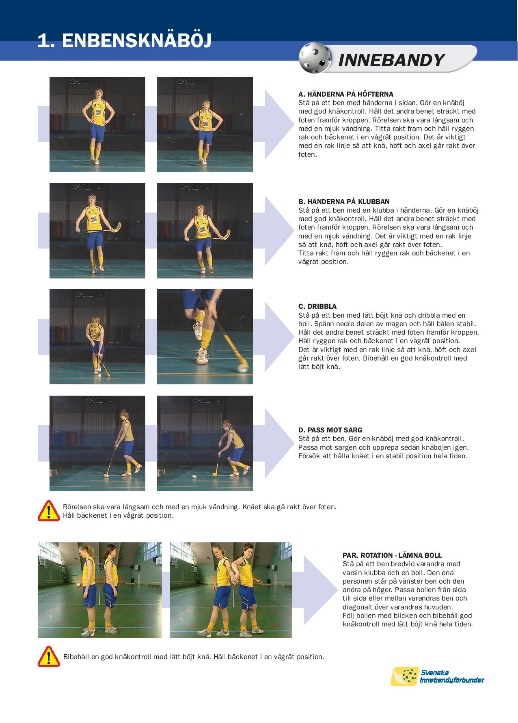 | 3×8-15 reps |
| Partner exercise | Both teammates stand on one leg with backs against each other. Hand over the floorball in different directions  **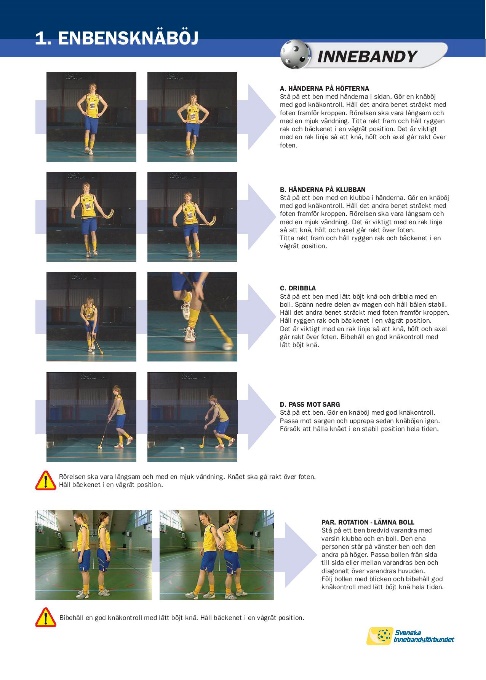** | 3×8-15 reps |

| Pelvic lift | Supine position; lift the pelvis from ground to neutral position while keeping the back straight | |
| --- | --- | --- |
| Level A | Both feet on the ground and hands across the chest  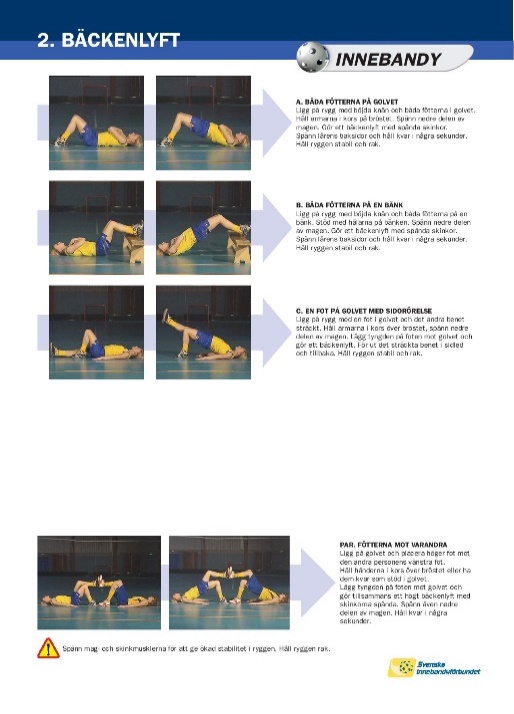 | 3×8-15 reps |
| Level B | Feet on a bench and hands across the chest  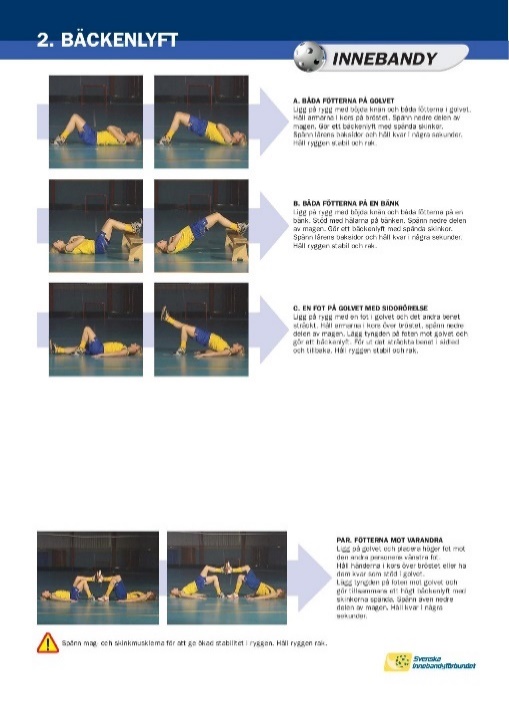 | 3×8-15 reps |
| Level C | One foot on the ground and the contralateral leg fixed straight, arms on the ground alongside the body. Lift the pelvis and abduct the straight leg  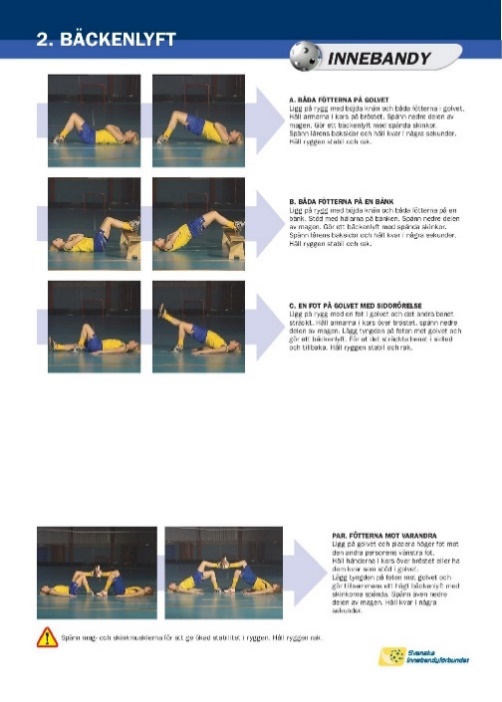 | 3×8-15 reps |
| Partner exercise | Supine position with feet towards each other. One foot on the ground and contralateral foot against the teammates foot  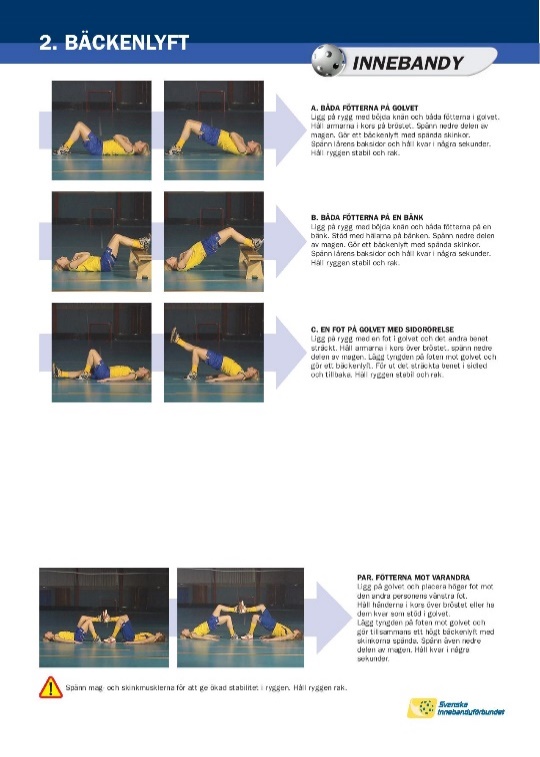 | 3×8-15 reps |

| Two legged knee squat | Slow movement with smooth turn, back straight, and feet shoulder-width apart, pointing forwards and with soles in contact with the ground. The trunk should be upright. | |
| --- | --- | --- |
| Level A | Hands on hips  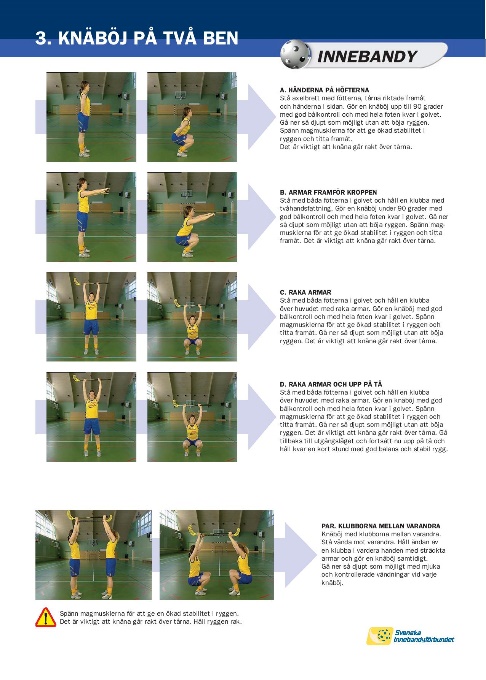 | 3×8-15 reps |
| Level B | Hold stick in front of the body with straight arms  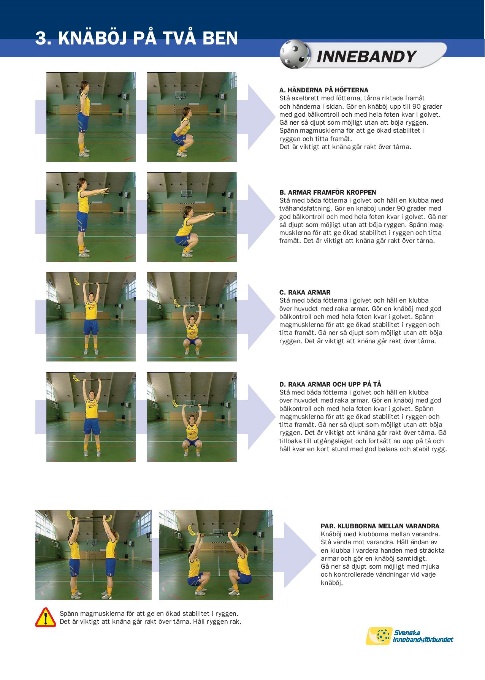 | 3×8-15 reps |
| Level C | Hold stick over the head with straight arms  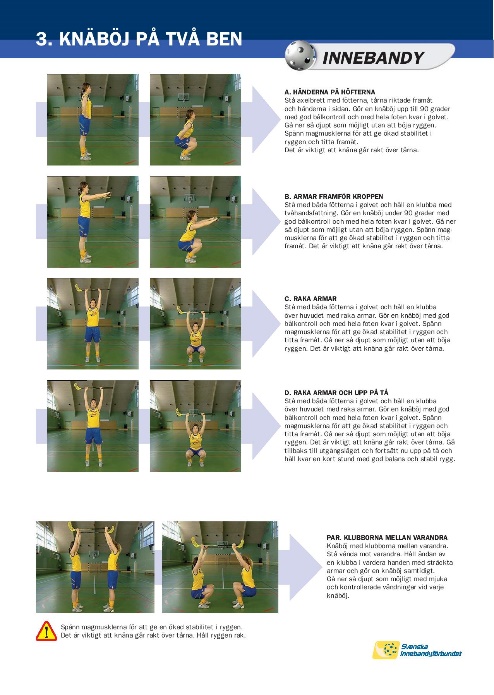 | 3×8-15 reps |
| Level D | Same as level C but continue movement and rise up on the toes after returning to the starting position and stay in the elevated position briefly  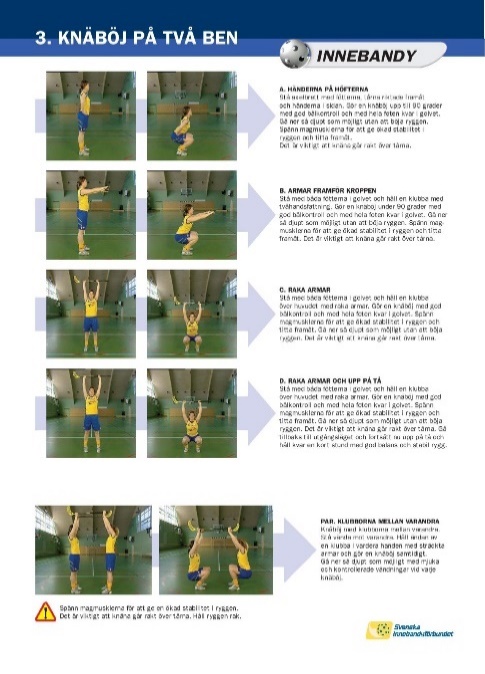 | 3×8-15 reps |
| Partner exercise | Teammate stands in front of you, facing towards you. Hold two sticks, one in each hand  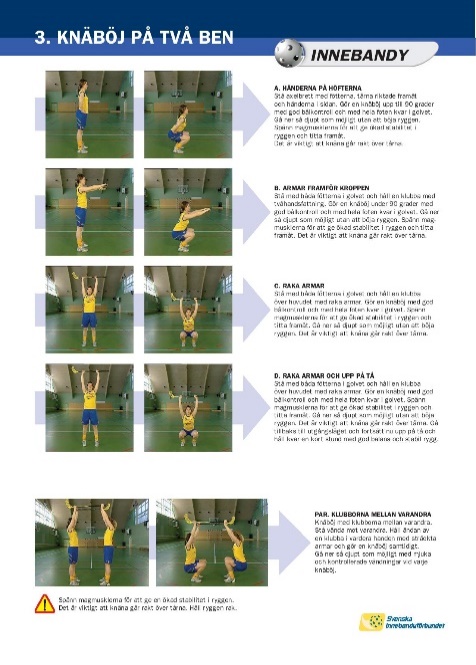 | 3×8-15 reps |

| The bench | Lift body and keep it in a straight line. Elbows placed beneath the shoulders with 90° flexion of the shoulders and elbows | |
| --- | --- | --- |
| Level A | Prone position; support on knees and on lower arms with elbows kept beneath the shoulders  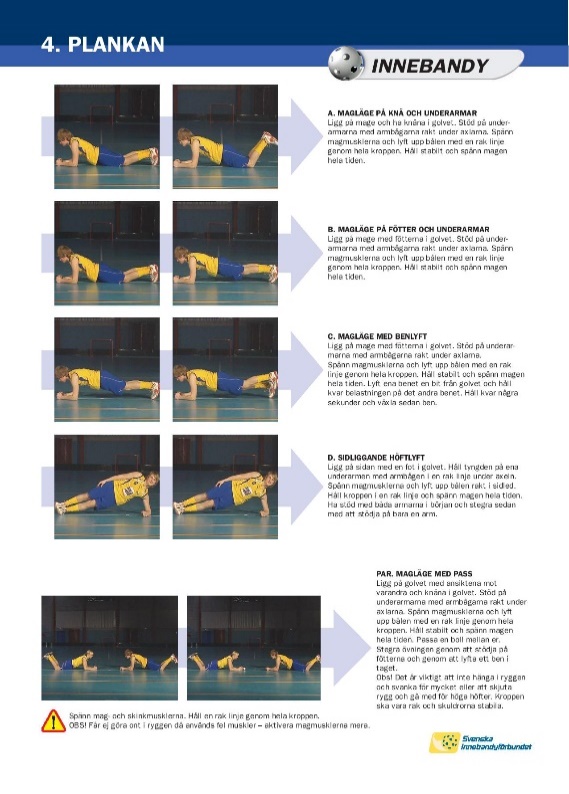 | 15-30 s |
| Level B | Same as level A but with support on the tip of the feet  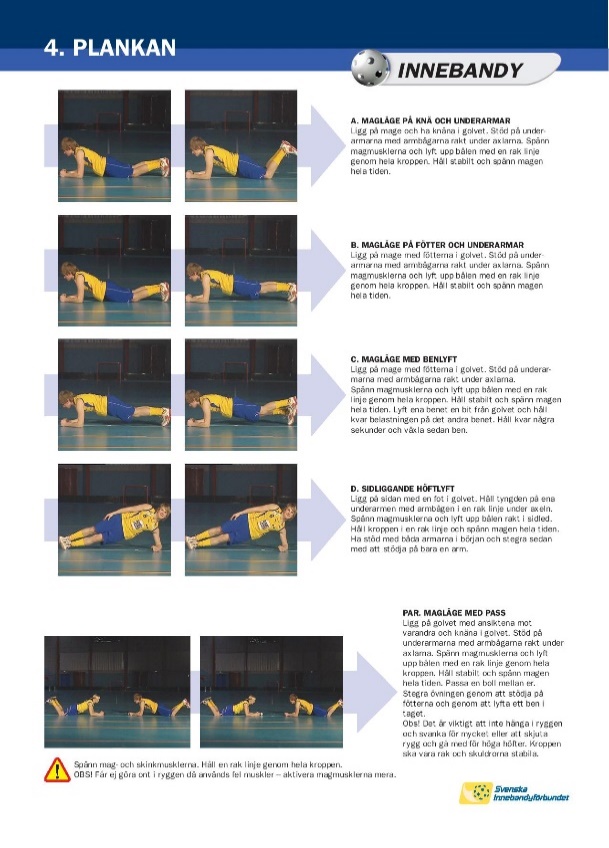 | 15-30 s |
| Level C | Same as level B, but raise one foot at a time up and down; alternate sides  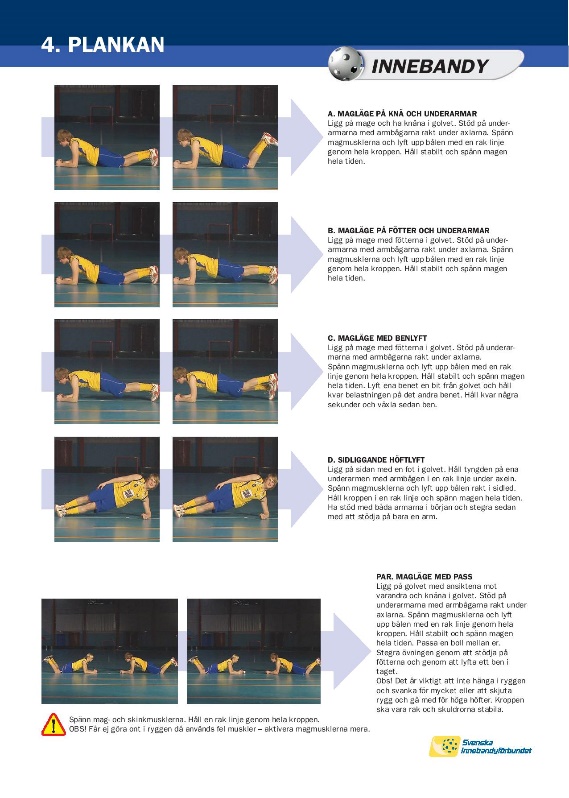 | 15-30 s |
| Level D | Lie sideways with support on the foot and lower arm with the elbow beneath the shoulder and the other hand on the hip; lift the hip off the ground, stay briefly in that position with good control, slowly return to the starting position.  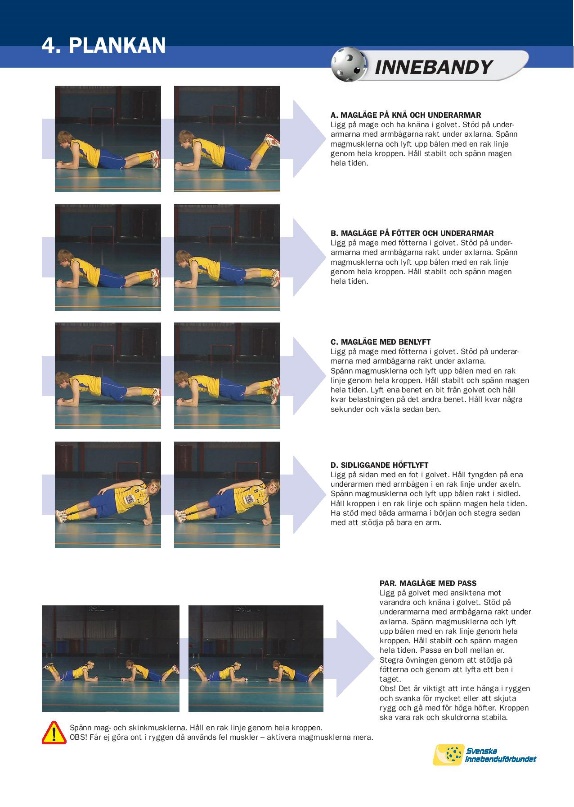 | 5-10 reps |
| Partner exercise | Same as level A but teammate face to face. Pass a floorball with the hand  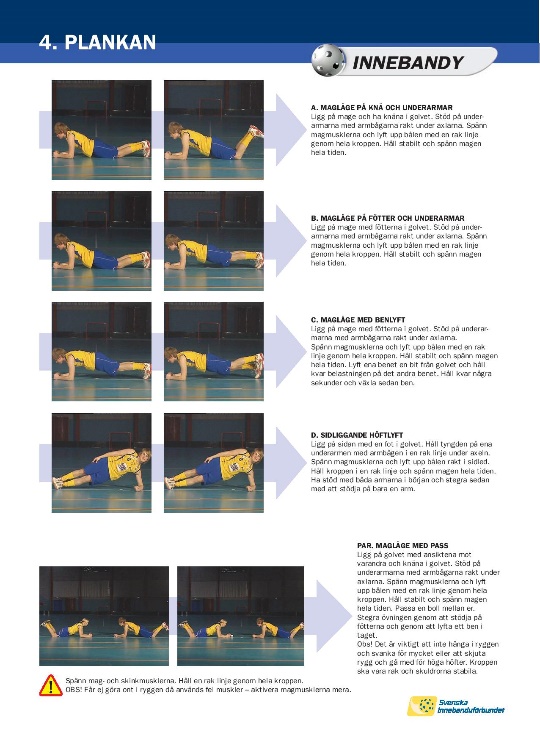 | 15-30 s |

| The lunge | Take a deep step forwards with a marked knee lift and soft landing; the rear knee should not touch the ground. Keep good knee-over-foot alignment. Trunk control maintained with minimum lateral movement and stable hips. Foot placed pointing forwards | |
| --- | --- | --- |
| Level A | Hands on the hips; move forwards with each step  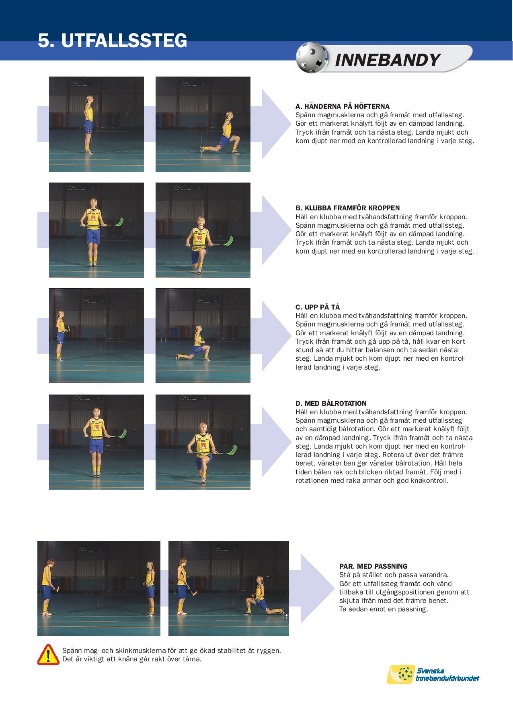 | 3×8-15 reps |
| Level B | Hold the stick in front of the body; move forwards with each step  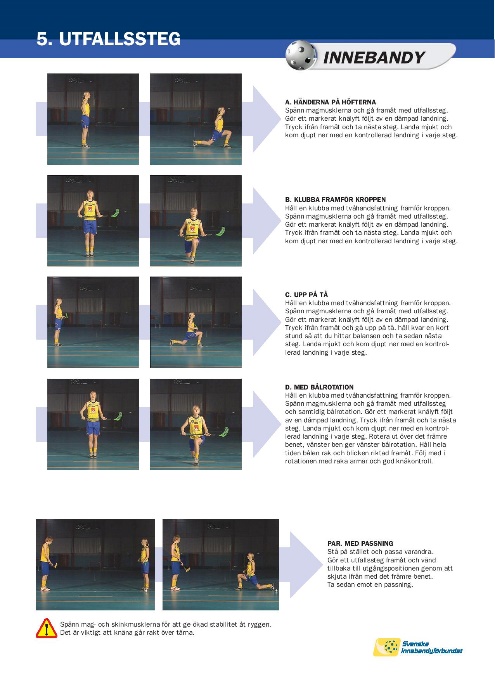 | 3×8-15 reps |
| Level C | Same as level B but continue the movement and rise up on the toes between each lunge  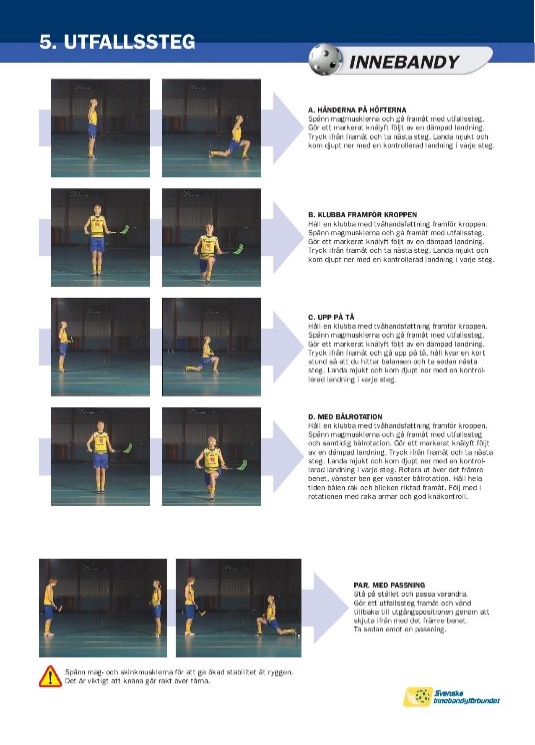 | 3×8-15 reps |
| Level D | Same as level B, rotate the upper body while stepping forwards and position the stick laterally to the front leg; move forwards with each step and alternate sides  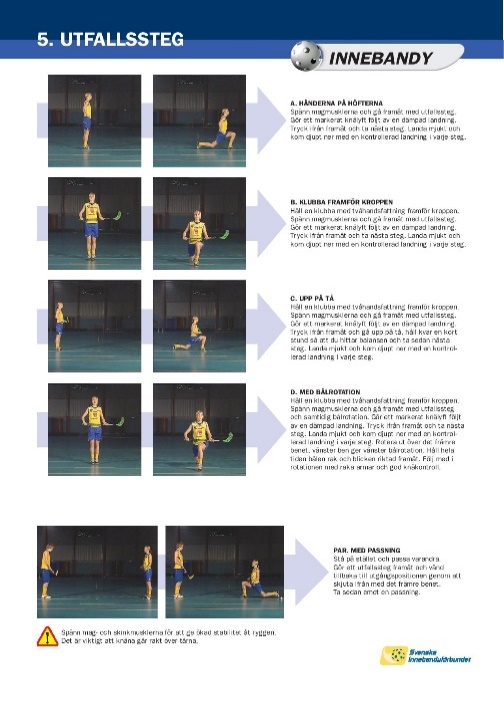 | 3×8-15 reps |
| Partner exercise | Teammate stands in front of you 5 m away; pass the floorball, perform a forward lunge, and receive a pass back  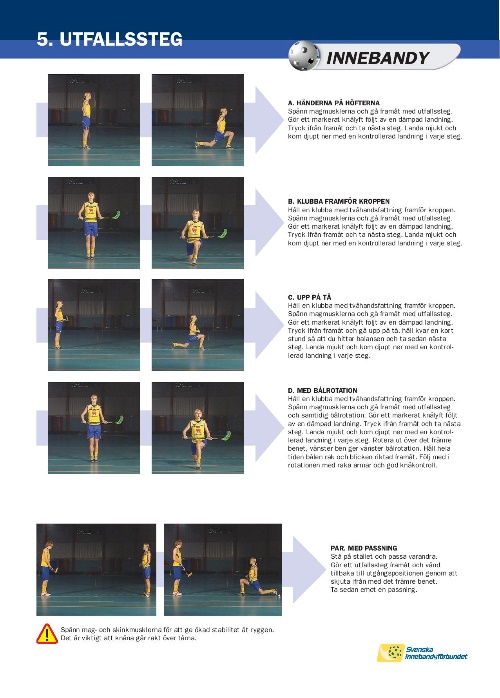 | 3×8-15 reps |

| Jump/landing | Jump with a controlled and soft landing; stay briefly in the landing position. Keep good knee-over-foot alignment. Trunk control maintained with minimal motion. Knee control maintained with minimal wobble sideways. | |
| --- | --- | --- |
| Level A | Stand on one leg with the knee slightly bent and hands on the hips; make a short forward jump and land on the same foot; then jump backwards to the starting position  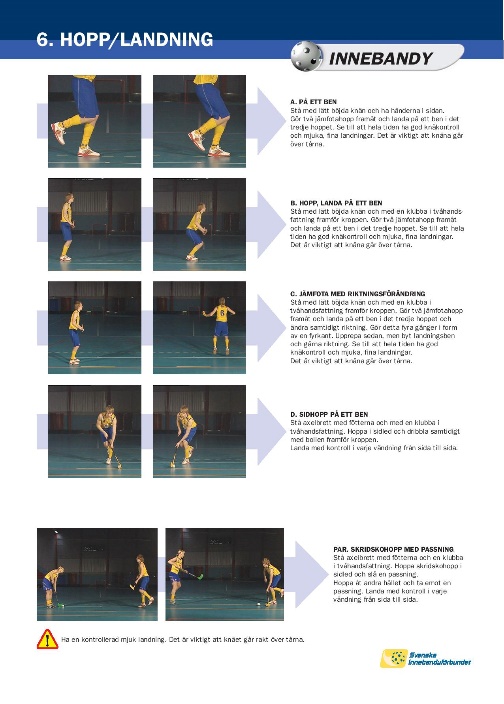 | 3×8-15 reps |
| Level B | Stand on both feet shoulder-width apart and hold a stick; make two forward jumps and land on one foot after the third jump with a controlled and soft landing; alternate sides  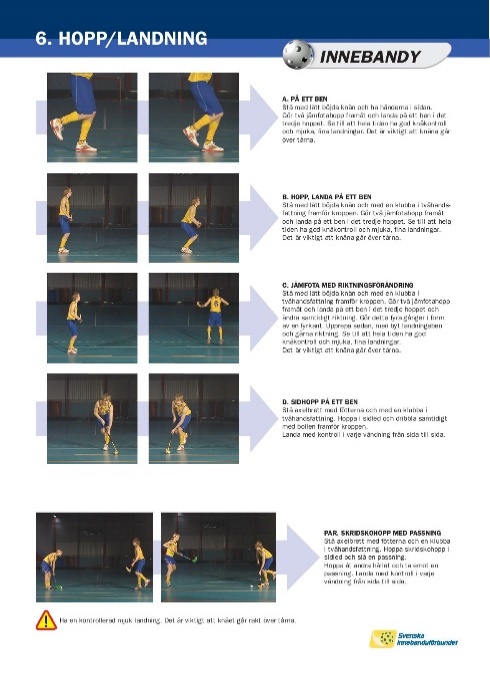 | 3×8-15 reps |
| Level C | Same as level B but rotate the body 90° before landing on one leg, alternate sides  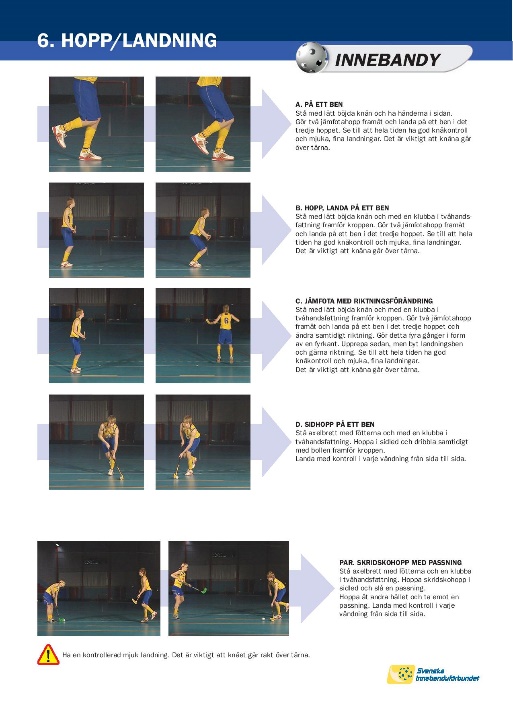 | 3×5 reps |
| Level D | Sideways jump on one foot and dribble with a ball and stick in front of you  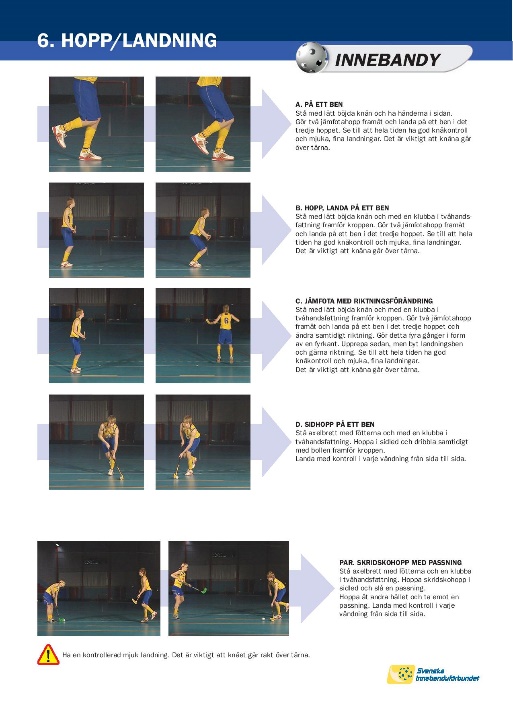 | 3×5 reps |
| Partner exercise | Teammate stands in front of you approximately 5 m away; make sideways jump on one foot and pass the floorball to your teammate. Jump back to the starting position and receive a pass back  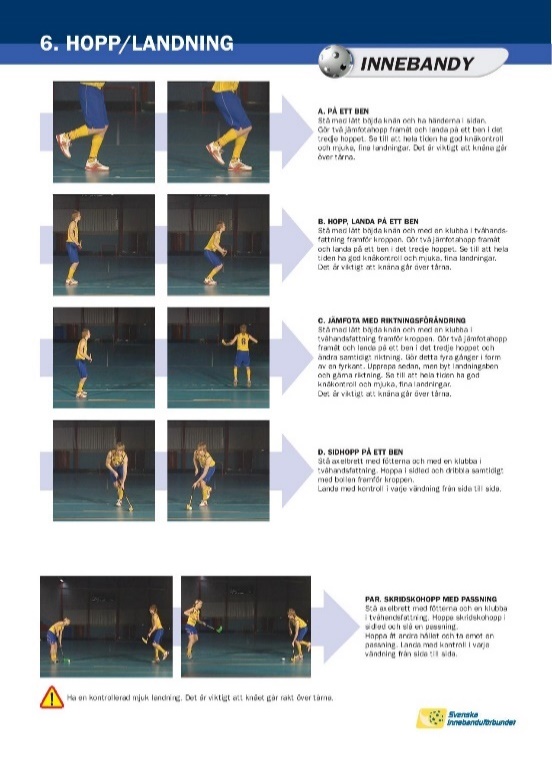 | 3×8-15 reps |
