# Supplementary material for: Perceptions, facilitators, and barriers regarding use of the injury prevention exercise programme Knee Control among players and coaches in youth floorball: a cross-sectional survey study
Source: BMC Sports Sci Med Rehabil. 2023 Apr 13;15:56. doi: 10.1186/s13102-023-00660-0 (PMC10103405; doi:10.1186/s13102-023-00660-0)
Supplement: Supplementary file 2 — Additional file 2. Pre-intervention player survey. The survey in its entirety, not all questions are relevant in this paper [file 13102_2023_660_MOESM2_ESM.docx]

Additional file 2. Pre-intervention player survey

Name:

**Team:**

**Age:**

**Sex**

- **Male**
- **Female**

Female, have you had your first period?

- Yes
- No

How many years have you played floorball (excluding the current season)?

Are you active in any other sport besides floorball?

- No
- Yes, __________________

Do you attend a class with a sports profile at school?

- No
- Yes, __________________

How many times per week do you play floorball in your team (training and match)?

- 1 time/week
- 2 times/week
- 3 times/week
- 4 times/week
- ≥ 5 times/week

How many times per week do you practice floorball at school?

- Not applicable
- 1 time/week
- 2 times/week
- 3 times/week
- 4 times/week
- ≥ 5 times/week

How many times per week do you participate in another sport (training and match)?

- 1 time/week
- 2 times/week
- 3 times/week
- 4 times/week
- ≥ 5 times/week

How do you perceive your current training volume (i.e., how much you exercise)? Circle an option.

| Low | 1 | 2 | 3 | 4 | 5 | 6 | 7 | High |
| --- | --- | --- | --- | --- | --- | --- | --- | --- |
|  | Extremely | Quite | Slightly | Neither | Slightly | Quite | Extremely |  |

How significantly have the following factors impacted your interest and motivation to participate in floorball?

|  | Low significance | | |  |  | | | |  | High significance | |
| --- | --- | --- | --- | --- | --- | --- | --- | --- | --- | --- | --- |
| Mother | 1 | 2 | 3 | | | 4 | 5 | 6 | | | 7 |
| Father | 1 | 2 | 3 | | | 4 | 5 | 6 | | | 7 |
| Sibling | 1 | 2 | 3 | | | 4 | 5 | 6 | | | 7 |
| Friends | 1 | 2 | 3 | | | 4 | 5 | 6 | | | 7 |
| Coach | 1 | 2 | 3 | | | 4 | 5 | 6 | | | 7 |
| Team | 1 | 2 | 3 | | | 4 | 5 | 6 | | | 7 |
| Club Environment | 1 | 2 | 3 | | | 4 | 5 | 6 | | | 7 |
| Proximity to the training facility | 1 | 2 | 3 | | | 4 | 5 | 6 | | | 7 |
| Your sporting success | 1 | 2 | 3 | | | 4 | 5 | 6 | | | 7 |
| Other:__________________________________________ | | | | | |  |  |  | | |  |

How do you currently experience your floorball participation?

|  | Do not agree | | |  |  | | | |  | | Agree |
| --- | --- | --- | --- | --- | --- | --- | --- | --- | --- | --- | --- |
| Thrives with teammates | 1 | 2 | 3 | | | 4 | 5 | 6 | | 7 | |
| Thrives with coaches | 1 | 2 | 3 | | | 4 | 5 | 6 | | 7 | |
| I have developed as a player the last year | 1 | 2 | 3 | | | 4 | 5 | 6 | | 7 | |
| I have performed well in floorball the last year | 1 | 2 | 3 | | | 4 | 5 | 6 | | 7 | |

Do you use floorball protective eyewear (goggles)?

- Yes, always for practice and games
- Only for practice
- Only for matches
- Sometimes
- Never

Have you used the *Knee Control* programme (or similar programme) before? The *Knee Control* programme is a training programme consisting of six different exercises: one-legged squat, pelvic lift, two-legged squat, the bench, lunges, jump and landing.

- Yes, regularly in the past year
- Yes, on and off in the past year
- No
- Do not know

Injury risk perceptions

In your opinion, how serious are the following types of injuries?

|  | Not at all serious | |  | Moderately serious | |  | Very serious |
| --- | --- | --- | --- | --- | --- | --- | --- |
| Ankle sprain | 1 | 2 | 3 | 4 | 5 | 6 | 7 |
| Knee sprain | 1 | 2 | 3 | 4 | 5 | 6 | 7 |
| Muscle strain | 1 | 2 | 3 | 4 | 5 | 6 | 7 |
| Broken bone | 1 | 2 | 3 | 4 | 5 | 6 | 7 |
| Cut or scrape | 1 | 2 | 3 | 4 | 5 | 6 | 7 |
| Bruise | 1 | 2 | 3 | 4 | 5 | 6 | 7 |
| Concussion | 1 | 2 | 3 | 4 | 5 | 6 | 7 |
| Eye injury | 1 | 2 | 3 | 4 | 5 | 6 | 7 |
| Dental injury | 1 | 2 | 3 | 4 | 5 | 6 | 7 |

**I expect I will sustain an injury sometime during this season…**

| **Unlikely** | **1** | **2** | **3** | **4** | **5** | **6** | **7** | **Likely** |
| --- | --- | --- | --- | --- | --- | --- | --- | --- |
|  | **Extremely** | **Quite** | **Slightly** | **Neither** | **Slightly** | **Quite** | **Extremely** |  |

**Many sports injuries are preventable with the help of training or protective equipment...**

| **False** | **1** | **2** | **3** | **4** | **5** | **6** | **7** | **True** |
| --- | --- | --- | --- | --- | --- | --- | --- | --- |
|  | **Extremely** | **Quite** | **Slightly** | **Neither** | **Slightly** | **Quite** | **Extremely** |  |
