# Supplementary material for: Perceptions, facilitators, and barriers regarding use of the injury prevention exercise programme Knee Control among players and coaches in youth floorball: a cross-sectional survey study
Source: BMC Sports Sci Med Rehabil. 2023 Apr 13;15:56. doi: 10.1186/s13102-023-00660-0 (PMC10103405; doi:10.1186/s13102-023-00660-0)
Supplement: Supplementary file 3 — Additional file 3. Pre-intervention coach survey. The survey in its entirety, not all questions are relevant in this paper [file 13102_2023_660_MOESM3_ESM.docx]

Additional file 3. Pre-intervention coach survey

Name:

**Team:**

Age:

Sex

- Male
- Female

How long have you been coaching floorball?
Since _____________ (year)

How long have you been coaching the current team?
Since _____________ (year)

Coach education in floorball
Specify level of education

Questions about injury risks in floorball and the *Knee Control* Programme
(Circle the number, 1-7, that best matches your opinion)

**What is your opinion about the overall risk of injury in floorball?**

| **Low** | **1** | **2** | **3** | **4** | **5** | **6** | **7** | **High** |
| --- | --- | --- | --- | --- | --- | --- | --- | --- |
|  | **Extremely** | **Quite** | **Slightly** | **Neither** | **Slightly** | **Quite** | **Extremely** |  |

In your opinion, how serious are the following types of injuries?

|  | Not at all serious | |  | Moderately serious | |  | Very serious |
| --- | --- | --- | --- | --- | --- | --- | --- |
| Ankle sprain | 1 | 2 | 3 | 4 | 5 | 6 | 7 |
| Knee sprain | 1 | 2 | 3 | 4 | 5 | 6 | 7 |
| Muscle strain | 1 | 2 | 3 | 4 | 5 | 6 | 7 |
| Broken bone | 1 | 2 | 3 | 4 | 5 | 6 | 7 |
| Cut or scrape | 1 | 2 | 3 | 4 | 5 | 6 | 7 |
| Bruise | 1 | 2 | 3 | 4 | 5 | 6 | 7 |
| Concussion | 1 | 2 | 3 | 4 | 5 | 6 | 7 |
| Eye injury | 1 | 2 | 3 | 4 | 5 | 6 | 7 |
| Dental injury | 1 | 2 | 3 | 4 | 5 | 6 | 7 |

**In general, how preventable do you think floorball injuries are?**

| **Not preventable** | **1** | **2** | **3** | **4** | **5** | **6** | **7** | **Preventable** |
| --- | --- | --- | --- | --- | --- | --- | --- | --- |
|  | **Extremely** | **Quite** | **Slightly** | **Neither** | **Slightly** | **Quite** | **Extremely** |  |

**My knowledge about preventing injuries in floorball is…**

| **Poor** | **1** | **2** | **3** | **4** | **5** | **6** | **7** | **Good** |
| --- | --- | --- | --- | --- | --- | --- | --- | --- |
|  | **Extremely** | **Quite** | **Fairly** | **Neither** | **Fairly** | **Quite** | **Extremely** |  |

**In your opinion, what would happen to a floorball player’s overall risk of injury if he/she participated in injury prevention training?**

| **Decrease** | **7** | **6** | **5** | **4** | **3** | **2** | **1** | **Increase** |
| --- | --- | --- | --- | --- | --- | --- | --- | --- |
|  | **Extremely** | **Quite** | **Slightly** | **Neither** | **Slightly** | **Quite** | **Extremely** |  |

**What do you think would happen to a floorball player's performance if he/she did injury prevention training regularly?**

| **Decrease** | **1** | **2** | **3** | **4** | **5** | **6** | **7** | **Increase** |
| --- | --- | --- | --- | --- | --- | --- | --- | --- |
|  | **Extremely** | **Quite** | **Slightly** | **Neither** | **Slightly** | **Quite** | **Extremely** |  |
