# Supplementary material for: Perceptions, facilitators, and barriers regarding use of the injury prevention exercise programme Knee Control among players and coaches in youth floorball: a cross-sectional survey study
Source: BMC Sports Sci Med Rehabil. 2023 Apr 13;15:56. doi: 10.1186/s13102-023-00660-0 (PMC10103405; doi:10.1186/s13102-023-00660-0)
Supplement: Supplementary file 4 — Additional file 4. Post-season player survey. The survey in its entirety, not all questions are relevant in this paper [file 13102_2023_660_MOESM4_ESM.docx]

Additional file 4. Post-season player survey

Name:

**Team:**

**My thoughts on training *Knee Control***

**After training *Knee Control* this season, I think my risk of injury has…**

| **Decreased** | **7** | **6** | **5** | **4** | **3** | **2** | **1** | **Increased** |
| --- | --- | --- | --- | --- | --- | --- | --- | --- |
|  | **Extremely** | **Quite** | **Slightly** | **Neither** | **Slightly** | **Quite** | **Extremely** |  |

**After training *Knee Control* this season, I have become faster, stronger and developed better balance…**

| **False** | **1** | **2** | **3** | **4** | **5** | **6** | **7** | **True** |
| --- | --- | --- | --- | --- | --- | --- | --- | --- |
|  | **Extremely** | **Quite** | **Slightly** | **Neither** | **Slightly** | **Quite** | **Extremely** |  |

**I have listened to my coach’s instructions on how to do the *Knee Control* exercises…**

| **Little** | **1** | **2** | **3** | **4** | **5** | **6** | **7** | **Much** |
| --- | --- | --- | --- | --- | --- | --- | --- | --- |
|  | **Extremely** | **Quite** | **Slightly** | **Neither** | **Slightly** | **Quite** | **Extremely** |  |

**I have been able to do all the exercises in the *Knee Control* programme correctly…**

| **Unsure** | **1** | **2** | **3** | **4** | **5** | **6** | **7** | **Sure** |
| --- | --- | --- | --- | --- | --- | --- | --- | --- |
|  | **Extremely** | **Quite** | **Slightly** | **Neither** | **Slightly** | **Quite** | **Extremely** |  |

**I have made 100% effort when we practised the *Knee Control* exercises…**

| **False** | **1** | **2** | **3** | **4** | **5** | **6** | **7** | **True** |
| --- | --- | --- | --- | --- | --- | --- | --- | --- |
|  | **Extremely** | **Quite** | **Slightly** | **Neither** | **Slightly** | **Quite** | **Extremely** |  |

**If my team uses *Knee Control* next season, I think it is…**

| **Bad** | **1** | **2** | **3** | **4** | **5** | **6** | **7** | **Good** |
| --- | --- | --- | --- | --- | --- | --- | --- | --- |
|  | **Extremely** | **Quite** | **Fairly** | **Neither** | **Fairly** | **Quite** | **Extremely** |  |

**Appraisal of *Knee Control* (several choices were possible)**

**Positive**

- **The exercises can reduce my risk of injury**
- **The exercises differ from the usual floorball training**
- **The structured warm-up with the same exercises in the same order every time**
- **I became better at performing *Knee Control* during the season**
- **The exercises can be made more difficult gradually**
- **I became a better player by training *Knee Control***
- **Do exercises together in the team or in pair**
- **The ball and/or the stick were used in some exercises**
- **Nothing, I did not like the programme**
- **Other:______________________________________**

**Negative**

- **I did not understand why I should do the exercises**
- **The exercises were too difficult/heavy**
- **The exercises were too easy**
- **The exercises were boring**
- **The programme was too long**
- **We had less time for the rest of the floorball training**
- **The exercises had nothing to do with floorball**
- **I feel pain when I do the exercises**
- **Nothing, I liked the programme**
- **Other:_______________________________________**
