# Supplementary material for: Perceptions, facilitators, and barriers regarding use of the injury prevention exercise programme Knee Control among players and coaches in youth floorball: a cross-sectional survey study
Source: BMC Sports Sci Med Rehabil. 2023 Apr 13;15:56. doi: 10.1186/s13102-023-00660-0 (PMC10103405; doi:10.1186/s13102-023-00660-0)
Supplement: Supplementary file 5 — Additional file 5. Post-season coach survey. The survey in its entirety, not all questions are relevant in this paper [file 13102_2023_660_MOESM5_ESM.docx]

## Additional file 5. Post-season coach survey

Name:

**Team:**

Questions about the use of *Knee Control* during the season

Reflect on your use of *Knee Control* during the past season and answer the following questions based on your normal use in your team.

On average, how many times per week did you play floorball?

- 1 time/week
- 2 times/week
- 3 times/week
- 4 times/week
- ≥ 5 times/week

How often did you use the *Knee Control* programme on average?

- <1 time/week
- 1 times/week
- 2 times/week
- 3 times/week
- >3 times/week

When, during the floorball training, did you use the *Knee Control* programme?

- Before the training session
- As part of the warm-up
- During the training session
- After the training session
- It varied

Do you also use the *Knee Control* programme at matches?

- Yes, the full programme mostly/always
- Yes, parts of the programme mostly/always
- Yes, the running warm-up mostly/always
- Occasionally
- No

How often did you use the different exercises in the programme? (tick the table, one answer per exercise)

|  | Never | Rarely | Mostly | Always |
| --- | --- | --- | --- | --- |
| Running warm-up |  |  |  |  |
| One legged knee squat |  |  |  |  |
| Pelvic lift |  |  |  |  |
| Two legged knee squat |  |  |  |  |
| The bench |  |  |  |  |
| The lunge |  |  |  |  |
| Jump/landing |  |  |  |  |

Each main exercise is available in five different progressions/variants at different levels of difficulty. How did you use the programme at training?

- We used the same exercise variant at each training session
- We selected different exercise variants across the season for variation
- We have chosen more difficult exercise variants over time
- We adapted exercise variants for the individual player
- We did like this: ________________________________________________________________

What exercise variants did you use most often? (mark with one or more crosses)

|  | Level A | Level B | Level C | Level D | Partner exercise |
| --- | --- | --- | --- | --- | --- |
| One legged knee squat |  |  |  |  |  |
| Pelvic lift |  |  |  |  |  |
| Two legged knee squat |  |  |  |  |  |
| The bench |  |  |  |  |  |
| The lunge |  |  |  |  |  |
| Jump/landing |  |  |  |  |  |

How many rounds (sets) did you practice the same exercise? ____________________ rounds

How much time did you spend on the *Knee Control* programme during a training session? ________min

Is there anything you want to add regarding the team's training with *Knee Control*?
(open question)

**Questions about your thoughts on injury prevention and *Knee Control***

**In general, how preventable do you think floorball injuries are?**

| **Not preventable** | **1** | **2** | **3** | **4** | **5** | **6** | **7** | **Preventable** |
| --- | --- | --- | --- | --- | --- | --- | --- | --- |
|  | **Extremely** | **Quite** | **Slightly** | **Neither** | **Slightly** | **Quite** | **Extremely** |  |

**My knowledge about preventing injuries in floorball is…**

| **Poor** | **1** | **2** | **3** | **4** | **5** | **6** | **7** | **Good** |
| --- | --- | --- | --- | --- | --- | --- | --- | --- |
|  | **Extremely** | **Quite** | **Fairly** | **Neither** | **Fairly** | **Quite** | **Extremely** |  |

**My practical ability to use *Knee Control* with my team is...**

| **Poor** | **1** | **2** | **3** | **4** | **5** | **6** | **7** | **Good** |
| --- | --- | --- | --- | --- | --- | --- | --- | --- |
|  | **Extremely** | **Quite** | **Fairly** | **Neither** | **Fairly** | **Quite** | **Extremely** |  |

**In your opinion, what has happened to the floorball players’ overall risk of injury after participating in injury prevention training?**

| **Decrease** | **7** | **6** | **5** | **4** | **3** | **2** | **1** | **Increase** |
| --- | --- | --- | --- | --- | --- | --- | --- | --- |
|  | **Extremely** | **Quite** | **Slightly** | **Neither** | **Slightly** | **Quite** | **Extremely** |  |

**In your opinion, what has happened to the floorball players’ performance after participating in injury prevention training?**

| **Decrease** | **1** | **2** | **3** | **4** | **5** | **6** | **7** | **Increase** |
| --- | --- | --- | --- | --- | --- | --- | --- | --- |
|  | **Extremely** | **Quite** | **Slightly** | **Neither** | **Slightly** | **Quite** | **Extremely** |  |

**Appraisal of *Knee Control***

***Knee Control* is floorball-specific…**

| **False** | **1** | **2** | **3** | **4** | **5** | **6** | **7** | **True** |
| --- | --- | --- | --- | --- | --- | --- | --- | --- |
|  | **Extremely** | **Quite** | **Slightly** | **Neither** | **Slightly** | **Quite** | **Extremely** |  |

***Knee Control* takes too much time…**

| **False** | **1** | **2** | **3** | **4** | **5** | **6** | **7** | **True** |
| --- | --- | --- | --- | --- | --- | --- | --- | --- |
|  | **Extremely** | **Quite** | **Slightly** | **Neither** | **Slightly** | **Quite** | **Extremely** |  |

***Knee Control* contains appropriate variation and progression for our team…**

| **False** | **1** | **2** | **3** | **4** | **5** | **6** | **7** | **True** |
| --- | --- | --- | --- | --- | --- | --- | --- | --- |
|  | **Extremely** | **Quite** | **Slightly** | **Neither** | **Slightly** | **Quite** | **Extremely** |  |

***Knee Control* can be used over several seasons in our team…**

| **False** | **1** | **2** | **3** | **4** | **5** | **6** | **7** | **True** |
| --- | --- | --- | --- | --- | --- | --- | --- | --- |
|  | **Extremely** | **Quite** | **Slightly** | **Neither** | **Slightly** | **Quite** | **Extremely** |  |

**Are you planning to prioritise injury prevention training in the form of *Knee Control* next season?**

| **Uncertain** | **1** | **2** | **3** | **4** | **5** | **6** | **7** | **Certain** |
| --- | --- | --- | --- | --- | --- | --- | --- | --- |
|  | **Extremely** | **Quite** | **Slightly** | **Neither** | **Slightly** | **Quite** | **Extremely** |  |

| **Facilitators and barriers** |
| --- |

**The following facilitators can help me perform *Knee Control* with my team at every training session in the next season (open answer)**

**The following barriers can make it difficult for me to perform *Knee Control* with my team at every training session in the next season (open answer)**
