# Supplementary material for: Perceptions, facilitators, and barriers regarding use of the injury prevention exercise programme Knee Control among players and coaches in youth floorball: a cross-sectional survey study
Source: BMC Sports Sci Med Rehabil. 2023 Apr 13;15:56. doi: 10.1186/s13102-023-00660-0 (PMC10103405; doi:10.1186/s13102-023-00660-0)
Supplement: Supplementary file 6 — Additional file 6. Tables S1-S3. Sex-separated results for both players and coaches. [file 13102_2023_660_MOESM6_ESM.pdf]

Additional file 6

Table 1.	Players' risk perceptions, outcome expectancies and action self-efficacy of *Knee Control* assessed pre-intervention and post-season, sex-separated results	
	**Male** ***n*=153**	**Female** ***n*=93**
**Risk perceptions**		
Pre-intervention		
I expect I will sustain an injury sometime during this season… (1 likely–7 unlikely)	4 (2)[a]	4 (2)[b]
**Outcome expectancies**		
Pre-intervention		
Many sports injuries are preventable with the help of training or protective equipment... (1 false–7 true)	6 (0)[a]	6 (2)[c]
Post-season		
After training *Knee Control* this season, I think my risk of injury has… (1 increased– 7 decreased)	5 (2)[d]	5 (2)
After training *Knee Control* this season, I have become faster, stronger and developed better balance… (1 false – 7 true)	5 (1)[d]	5 (2)
**Action self-efficacy**		
Post-season		
I have listened to my coach's instructions on how to do the *Knee Control* exercises… (1 little – 7 much)	6 (1)[d]	6 (1)
I have been able to do all the exercises in the *Knee Control* programme correctly… (1 unsure – 7 sure)	6 (2)[d]	6 (2)
I have made a 100% effort when we practiced the *Knee Control* exercises… (1 false – 7 true)	6 (1)[d]	6 (1)

All results are presented as median with interquartile range in brackets. “Extremely” to be added to all anchors in the Likert scale.

[a] Missing for 6 players

[b] Missing for 4 players

[c] Missing for 5 players

[d] Missing for 1 player

Table 2.	Outcome expectancies, action self-efficacy and appraisal of *Knee Control* separated for coaches for male (*n*=23) versus female (*n*=12) teams			
	**Pre-intervention**		**Post-season**	
	**Male**	**Female**	**Male**	**Female**
**Outcome expectancies**				
In general, how preventable do you think floorball injuries are? (1 not preventable – 7 preventable)	6 (1)	5.5 (1)	6 (1)	6 (1)
In your opinion, what would/has happen/ed to a floorball player's overall risk of injury if he/she participated in injury prevention training? (1 increase– 7 decrease)	5 (4)	5.5 (4)	3 (3)	5 (2)
What do you think would/has happen/ed to a floorball player's performance if he/she did injury prevention training regularly? (1 decrease– 7 increase)	5 (1)	5.5 (1)	5 (2)	5 (2)
**Action self-efficacy**				
My knowledge about preventing injuries in floorball is… (1 poor – 7 good)	4 (2)	3.5 (2)	5 (1)	5 (1)
My practical ability to use *Knee Control* with my team is... (1 poor–7 good)			5 (1)[a]	5 (2)
**Appraisal of *Knee Control***				
Knee Control is floorball specific… (1 false – 7 true)			5 (2)	5 (1)
Knee Control takes too much time… (1 false – 7 true)			4 (3)	3 (3)
Knee Control contains appropriate variation and progression for our team… (1 false – 7 true)			5 (1)	5 (1)
Knee Control can be used over several seasons in our team… (1 false – 7 true)			6 (1)	6 (1)

All results are presented as median with interquartile range in brackets. "Extremely" to be added to all anchors in the Likert scale, e.g., 1 extremely not preventable – 7 extremely preventable.

[a] Missing for 1 coach

Table 3. Players' appraisals of *Knee Control*, in total and sex-separated results			
	**Total (n=246)**	**Male (n=153)**	**Female (n=93)**
**Positive**			
The exercises can reduce my risk of injury, n (%)	216 (88)	133 (87)	83 (89)
I became better at performing *Knee Control* during the season, n (%)	177 (72)	111 (73)	66 (71)
Do exercises together in the team or in pair, n (%)	117 (48)	68 (44)	49 (53)
The ball and/or the stick were used in some exercises, n (%)	102 (41)	60 (39)	42 (45)
The structured warm-up with the same exercises in the same order every time, n (%)	99 (40)	60 (39)	39 (42)
The exercises differ from the usual floorball training, n (%)	84 (34)	51 (33)	33 (35)
The exercises can be made more difficult gradually, n (%)	80 (33)	46 (30)	34 (37)
I became a better player by training *Knee Control*, n (%)	78 (32)	46 (30)	32 (34)
Nothing, I did not like the programme, n (%)	18 (7)	15 (10)	3 (3)
I did not understand why I should do the exercises, n (%)[a]	16 (7)	12 (8)	4 (4)
**Negative**			
We had less time for the rest of the floorball training, n (%)[a]	103 (43)	63 (43)	40 (43)
The exercises were boring, n (%)[a]	96 (40)	65 (44)	31 (33)
Nothing, I liked the programme, n (%)[a]	59 (25)	35 (24)	24 (26)
I feel pain when I do the exercises, n (%)[a]	58 (24)	30 (20)	28 (30)
The programme was too long, n (%)[a]	47 (20)	30 (20)	17 (18)
The exercises were too easy, n (%)[a]	36 (15)	25 (17)	11 (12)
The exercises had nothing to do with floorball, n (%)[a]	29 (12)	23 (16)	6 (6)
The exercises were too difficult/heavy, n (%)[a]	10 (4)	7 (5)	3 (3)

[a] Missing for 6 male players
